# Vitamin D_3_ at 50x AI Attenuates the Decline in Paw Grip Endurance, but Not Disease Outcomes, in the G93A Mouse Model of ALS, and Is Toxic in Females

**DOI:** 10.1371/journal.pone.0030243

**Published:** 2013-02-06

**Authors:** Alexandro Gianforcaro, Jesse A. Solomon, Mazen J Hamadeh

**Affiliations:** 1 School of Kinesiology and Health Science, Faculty of Health, York University, Toronto, Ontario, Canada; 2 Muscle Health Research Centre, York University, Toronto, Ontario, Canada; 3 Department of Pediatrics, McMaster University, Hamilton, Ontario, Canada; University of Florida, United States of America

## Abstract

**Background:**

We previously demonstrated that dietary vitamin D_3_ at 10x the adequate intake (AI) attenuates the decline in functional capacity in the G93A mouse model of ALS. We hypothesized that higher doses would elicit more robust changes in functional and disease outcomes.

**Objective:**

To determine the effects of dietary vitamin D_3_ at 50xAI on functional outcomes (motor performance, paw grip endurance) and disease severity (clinical score), as well as disease onset, disease progression and lifespan in the transgenic G93A mouse model of ALS.

**Methods:**

Starting at age 25 d, 100 G93A mice (55 M, 45 F) were provided *ad libitum* with either an adequate (AI; 1 IU D_3_/g feed) or high (HiD; 50 IU D_3_/g feed) vitamin D_3_ diet.

**Results:**

HiD females consumed 9% less food corrected for body weight vs. AI females (P = 0.010). HiD mice had a 12% greater paw grip endurance over time between age 60–141 d (P = 0.015), and a 37% greater score during disease progression (P = 0.042) vs. AI mice. Although HiD females had a non-significant 31% greater CS prior to disease onset vs. AI females, they exhibited a significant 20% greater paw grip endurance AUC (P = 0.020) when corrected for clinical score.

**Conclusion:**

Dietary D_3_ supplementation at 50x the adequate intake attenuated the decline in paw grip endurance, but did not influence age at disease onset, hindlimb paralysis or endpoint in the transgenic G93A mouse model of ALS. Furthermore, females may have reached the threshold for vitamin D_3_ toxicity as evidence by reduced food intake and greater disease severity prior to disease onset.

## Introduction

Amyotrophic lateral sclerosis (ALS; also known as Lou Gehrig's disease) is a neurodegenerative/neuromuscular disease characterized by progressive degeneration of motor neurons in the central nervous system (CNS), resulting in muscle weakness followed by paralysis [1]. Death ensues due to respiratory failure with a median survival rate of 3–5 years after the first appearance of symptoms [1]. With a prevalence of about 4 in 100,000, approximately 10% of ALS cases are known to be due to genetically inherited mutations (familial ALS), whereas the vast majority of ALS cases are of unknown cause (sporadic ALS) [2]. Familial and sporadic ALS are clinically and pathologically similar [2]. The pathophysiology is multi-faceted and involves oxidative stress, inflammation, glutamate excitotoxicity, and neurodegeneration [3].

Different dietary interventions have been studied in human and rodent models of ALS and are reviewed by Patel and Hamadeh [4]. Interestingly, despite the breadth of literature demonstrating the benefits of caloric restriction across many species and phyla, both short-term and long-term caloric restriction hasten disease onset and shorten lifespan in the G93A mouse [5]–[7]. The only FDA-approved treatment in humans to-date is the anti-glutamatergic drug Riluzole which prolongs median survival by 2–3 mo [8]. In contrast, vitamin D attenuates several mechanisms involved in ALS disease pathology, such as 1) oxidative stress (increased liver and kidney antioxidant enzyme activity by up to 4.4 fold, and decreased lipid peroxidation by up to 46%) [9], 2) inflammation (increased serum IL-10 by 43%, while inhibiting rise in TNF-α) [10], 3) glutamate excitotoxicity (increased neuronal survival *in vitro* by 17% and 50% with 10 nM and 100 nM calcitriol, respectively) [11] and 4) neuronal death while increasing expression of neurotrophic factors [12]–[15]. Indeed, tissues directly related to ALS pathology express the vitamin D receptor (brain, spinal cord and skeletal muscle) and/or the enzyme 25(OH)D_3_-1α-hydroxylase [1α(OH)ase] that converts the less active 25(OH)D_3_ (calcidiol) into the fully active 1,25(OH)_2_D_3_ (calcitriol) form of the vitamin (brain and spinal cord) [16]–[18]. The above findings suggest that supplementing vitamin D_3_ or its metabolites may attenuate multiple mechanisms involved in ALS pathology.

The principal concern when supplementing with very high doses of vitamin D_3_ is the threat of toxicity as expressed by hypercalcemia [19], or a decrease in food intake and/or body weight [20], [21]. In female Balb/c mice, i.p. injections of 63 IU/d (∼2.7 IU/g b.wt./d), 252 IU/d (∼11 IU/g b.wt./d) and 440 IU/d (∼19 IU/g b.wt./d) of vitamin D_3_ over 6 d allowed for 100% survival 3 wk post-injection, whereas 630 IU D_3_/d (∼27 IU D_3_/g b.wt./d) yielded 70% survival [22]. Based on increased serum calcium concentration and decreased body weight, 200 IU dietary vitamin D_3_/d (∼9 IU D_3_/g b.wt./d) induced toxicity in male B10.PL mice [21]. This was probably due to the high calcium content in the feed (1.0%), especially since calcium increases the biological potency of vitamin D_3_ metabolites [23]. In contrast, a separate study supplementing C57BL/6 mice with similar vitamin D_3_ and calcium showed no differences in body weights of supplemented mice compared to wild type controls at 12 wk, possibly due to the difference in murine strain [24]. Given these results, dietary intakes of vitamin D_3_ up to ∼9 IU D_3_/g b.wt./d, equivalent to ∼55 fold the adequate intake (AI), with a 0.5% calcium diet should be non-toxic in mice.

G93A mice transgenically overexpress the mutant human SOD1 gene and follow the same disease pattern as human ALS patients [25], [26], and can thus be used as a rodent model of ALS. We previously demonstrated that vitamin D_3_ supplementation at 10x the AI attenuates the rapid disease-induced decline in paw grip endurance (7%) and motor performance (22%) vs. mice consuming the AI (1 IU/g feed) in the transgenic G93A mouse model of ALS [27]. However, we detected no significant differences in disease severity and other disease outcomes such as age at disease onset, functional hind-limb paralysis or endpoint (the point at which the mouse is unable to right itself to sternum within 20 s) [27]. This could be due to a type-II error, however it is also possible that vitamin D_3_ at 10x the AI does not affect these disease outcomes in this model. We wondered whether a higher dose of dietary vitamin D_3_ would elicit significant differences in disease outcomes, as well as more robust changes in functional outcomes. Hence, the objective of this study was to examine the effects of higher levels of vitamin D_3_ supplementation (50 IU/g feed) vs. the adequate intake (1 IU/g feed) on functional and disease outcomes, in the transgenic G93A mouse model of ALS.

## Methods

### Ethical statement

The experimental protocol in this study followed the guidelines of the Canadian Council of Animal Care and was approved by York University Animal Research Ethics Board (protocol # 2007-9). All necessary steps were taken to minimize suffering and distress to the mice in the study.

### Animals

Male B6SJL-TgN(SOD1-G93A)1Gur hemizygous mice (No. 002726) were harem-bred with nonaffected female B6SJL control mice (No. 100012; Jackson Laboratory, Bar Harbor, ME). The presence of the human-derived G93A transgene was confirmed using polymerase chain reaction (PCR) amplification of DNA extracted from ear tissue as outlined by Sigma-Aldrich (XNAT REDExtract-N-Amp Tissue PCR Kit; XNAT-1KT). All breeding mice were housed 3 females per 1 male, and consumed Research Diet AIN-93G (1 IU D_3_/g feed; Research Diet, New Brunswick, NJ). All animals were housed individually at age 25 d in a 12 h light/dark cycle.

### Study design

One-hundred G93A mice (55 M, 45 F) consumed a diet containing an adequate amount of vitamin D_3_ (1 IU/g feed; Research Diet AIN-93G; Product # D10012G; Research Diets Inc, New Brunswick, NJ [28]) *ad libitum* after weaning (21 d). At age 25 d, mice were housed in individual cages and divided into one of two groups: 1) adequate vitamin D_3_ (AI; 1 IU D_3_/g feed; 30 M, 24 F) and 2) high vitamin D_3_ (HiD; 50 IU D_3_/g feed; 25 M, 21 F; product # D10030802; Research Diets Inc, New Brunswick, NJ) (Table 1). A subset of these mice (31 AI: 18 M, 13 F; and 28 HiD: 15 M, 13 F) were followed to endpoint, whereas the remaining 41 mice (23 AI: 12 M, 11 F; and 18 HiD: 10 M, 8 F) were sacrificed at age 113 d for *tibialis anterior*, *quadriceps* and brain harvesting. Preliminary results from this study had shown differences in clinical score (CS; disease severity) between HiD and AI mice at age 113 d.

**Table 1:** Nutrient content of the adequate intake (AI) and high (HiD) vitamin D_3_ diets.

Nutrient	Diet	
	AI	HiD
Energy (kcal/g)	4	4
Carbohydrate (%)	64	64
Protein (%)	20	20
Fat (%)	7	7
Vitamin D_3_ (IU/g)	1 a	50 a, b
Calcium (%)	0.5 c	0.5 c
Vitamin mix V10037 (mg/g)	10	10
Mineral mix S100022G (mg/g)	35	35

Diets provided by Research Diets (based on AIN-93G; New Brunswick, NJ; AI product # D10012G; HiD product # D08080101).

a , included in vitamin mix V10037 [55].

b , additional vitamin D_3_ was added to reach 50 IU/g feed.

c , included in mineral mix S100022G [56].

When mice reached a CS of 3.0, food and calorie-free gel (Harlan-Gel, Harlan Teklad, Madison, WI) were placed on the floor of the cage to fulfill the requirements of the ethics committee. The calorie-free gel contained synthetic polymers (WATER LOCK® superabsorbent polymer G-400, G- 430, G-500, G-530; 95% by weight) and methanol (4.5% by weight). All measurements were conducted by two researchers who were blinded to the diets. The intra-tester coefficients of variation (CV) for researcher #1 were 1.8% for body condition, 0.0% for ability to move and 1.1% for clinical score, whereas for researcher #2 the intra-tester CV were 1.8% for body condition, 0.0% for ability to move and 1.2% for clinical score. The inter-researcher CV was 0.0% for body condition, 1.06% for ability to move and 0.96% for clinical score.

### Food intake and body weight

Beginning at age 25 d, food intake and body weight were recorded twice per wk for all mice. When mice reached a clinical score of 3.0, body weight was recorded daily until endpoint.

### Body condition (BC)

Beginning at age 60 d, BC was recorded twice per wk until mice reached a clinical score of 3.0, thereafter BC was recorded daily until endpoint. Body condition followed a 5-point scale: 5 =  obese mice, 4 =  over-conditioned mice (spine is a continuous column and the vertebrae are palpable only with firm pressure), 3 =  well-conditioned mice (the vertebrae and dorsal pelvis are not prominent and are palpable with slight pressure), 2 =  under-conditioned mice (the segmentation of the vertebral column is evident and the dorsal pelvic bones are easily palpable), and 1 =  emaciated mice (the skeletal structure is extremely prominent and the vertebrae are distinctly segmented).

### Ability to move (ATM)

Beginning at age 60 d, ATM was recorded twice per wk until mice reached a clinical score of 3.0, thereafter ATM was recorded daily until endpoint. Ability to move followed a 5-point scale: 4 =  normal mobility, 3 =  moving with limited use of the hindlimbs, 2 =  moving with the use of the forelimbs, 1 =  moving only for a short period with the use of the forelimbs, and 0 =  not moving.

### Paw grip endurance (PaGE)

Beginning at age 60 d, PaGE was recorded 3 times every 10 d (measurements were separated by 2–3 days) until endpoint, using the modified hanging wire test [29], [30]. Animals were placed on a wire grid held at a height of ∼40 cm, the grid was gently shaken to cause the mouse to tighten its grip on the wires then inverted, and time recording commenced. The time was recorded until the mouse lost its grip in 4 limbs, for a maximum score of 180 s. This test was completed in triplicate, with the highest score used for analysis.

### Motor performance (MP)

Beginning at age 60 d, MP was recorded once every 10 d until endpoint using the rotarod test (AccuScan Instruments, Inc., Columbus, OH). Mice were placed on a rod (30 mm diameter at a height of 39 cm, covered with corrugated rubber to allow for traction) that rotated at a gradually increasing speed to 45 rpm over 60 s and remained at 45 rpm until the mouse could no longer stay on the rod. The rotarod apparatus was interfaced with a computer that initiated the test and recorded the competency score. Motion sensors located at the bottom of the rotarod chamber were activated when the mouse fell off the rod and, as a result, the computer ended the recording session. The test was performed in triplicate, with the highest score used for analysis.

### Clinical score (CS; disease severity)

Beginning at age 60 d, CS was recorded daily until endpoint and followed an 8-point scale based on signs of weakness exhibited by the mice in order to establish disease severity: 0 =  no evidence of disease, 1 =  shaking or splaying of the hindlimbs when suspended by the tail (an indication of weakness in the hindlimbs), 1.5 =  weakness in one hindlimb (compensation for footdrop), 2 =  weakness in both hindlimbs (change in gait; used as disease onset when attained on two consecutive days), 2.5 =  extreme weakness in one hindlimb (inability to dorsiflex), 3 =  extreme weakness in both hindlimbs, 3.5 =  functional paralysis in one hindlimb, 4 =  functional paralysis in both hindlimbs, and 5 =  mouse cannot right itself within 20 s after being placed on its side (considered as endpoint [31]). For all mice, CS was recorded prior to all other functional measurements.

### Tibialis anterior, quadriceps and brain

At age 113 d, 41 G93A mice were anesthetized with isoflurane gas and maintained under general anesthesia while tissues were collected. *Tibialis anterior, quadriceps* and brain were immediately removed and weighed.

### Statistical analysis

A three-way repeated measures ANOVA (between-subject factors: diet and sex; within-subject factor: time) was used to determine significant sex differences over time in absolute food intake, food intake corrected for body weight, body weight, BC, ATM, PaGE, MP and CS. A two-way repeated measures ANOVA (between-subject factor: diet; within-subject factor: time) was used to determine significant diet differences over time for BC, ATM, PaGE, MP and CS in three ways: 1) using data from the first day of testing (age 60 d) until the age at which the first group achieved a mean clinical score of 5 (endpoint; 141 d), 2) using data from the first day of testing (age 60 d) until the age at which the first group achieved a mean clinical score of 2 (disease onset; 105 d) (i.e. prior to disease onset), and 3) using data from the age at which the first group achieved a mean clinical score of 2 (disease onset; 105 d) until the age at which the first group achieved a mean clinical score of 5 (endpoint; 141 d) (i.e. during disease progression). Statistical analysis using data for 60–105 d and 60–113 d with and without harvest mice were not different, therefore we reported results for 60–105 d without harvest mice. In order to achieve maximum statistical power, statistical analysis was performed including all mice when possible [food intake (absolute and mg/g b.wt.), body weight and disease onset – CS2]. When ANOVA indicated significance, a Newman-Keuls post-hoc test was used to determine the source of difference. A Student's t-test was used to determine significant differences in disease onset (CS2), hindlimb paralysis (CS4), endpoint (CS5), number of days between CS2 to CS5 (disease progression) and area under the curve (AUC) scores between the diets (within sex and when sexes were combined). Statistical analyses for AUC scores for BC, ATM, PaGE, MP and CS were conducted in three ways: 1) using data from first day of testing (age 60 d) until CS5 specific for each mouse, 2) using data from age 60 d until CS2 specific for each mouse (i.e. prior to disease onset), and 3) using data from CS2 until CS5 specific for each mouse (i.e. during disease progression). AUC scores for each mouse were corrected for the number of days between age 60 d and CS5 (first approach), between age 60 d and CS2 (second approach), or between CS2 and CS5 (third approach). Analysis of data was performed between age 60 d and CS5 to investigate the effect of the HiD diet over the entire study period, between age 60d and CS2 to investigate the effect of the HiD diet prior to disease onset, and between CS2 and CS5 to investigate the effect of the HiD diet during disease progression. A logrank test was used to determine differences in the rate at which the groups reached CS2, CS4 and CS5.

A three-way repeated measures ANOVA (between-subject factors: diet and sex; within-subject factor: repeated measures) was also used to determine sex differences in the following correlations between BC, ATM, PaGE, and MP vs. CS; ATM, PaGE and MP vs. BC; PaGE and MP vs. ATM; as well as MP vs. PaGE. A two-way repeated measures ANOVA (between-subject factors: diet; within-subject factor: repeated measures) was used for the above correlations to determine diet differences within sex or when sexes were combined. In addition, AUC for the above correlations were subjected to a two-way ANOVA (diet and sex) to determine sex differences, or a Student's t-test to determine diet differences within sex or when sexes were combined. Using data from mice followed to endpoint, correlations between the AUC for anthropometric (BC), functional (ATM, PaGE and MP) and disease (CS, CS2, CS4, CS5 and disease progression) outcomes were conducted.

A Student's t-test was used to determine diet and sex differences in absolute and body weight-adjusted *tibialis anterior*, *quadriceps* and brain weights. Body weight-adjusted *tibialis anterior*, *quadriceps* and brain weights were correlated with age at CS2 for each tissue harvest mouse. Group means for body weight-adjusted *tibialis anterior*, *quadriceps* and brain weights were also correlated with the group means for age at CS4, CS5 and disease progression for endpoint mice within the same diet and sex. A two-tailed test was used for all statistical comparisons for outcome measures except for BC, ATM, PaGE and MP where a one-tailed test was used, because based on the scientific literature we *a priori* hypothesized that high vitamin D_3_ would improve functional outcomes (ATM, PaGE and MP). Our lab has recently demonstrated that vitamin D_3_ supplementation at 10 fold the AI delays the decline in paw grip endurance and motor performance in the G93A mouse [27]. Statistical analyses were performed using Statistica 6.0 Windows (version 6.0, StatSoft, Tulsa, OK). Significance was considered at P≤0.05, and trends were considered at 0.05<P≤0.15. Data are presented as means ± standard error of the mean (SEM).

## Results

### Food intake

Food intake was not significantly different between the diets (Table 2). AI mice had an average absolute food intake of 3.37±0.05 g/d (equivalent to 169.8≥3.0 mg/g b.wt./d), corresponding to an average vitamin D_3_ intake of 3.37 IU/d (0.170 IU/g b.wt./d) (Figure S1A and S1B). HiD mice had an average absolute food intake of 3.11±0.05 g/d (162.1±3.3 mg/g b.wt./d), corresponding to an average vitamin D_3_ intake of 155.5 IU/d (8.11 IU/g b.wt./d). Males (3.33±0.05 g/d) consumed 8% more absolute food vs. females (3.15±0.05 g/d; P = 0.014).

**Table 2:** Food intake, vitamin D_3_ intake and body weight of G93A mice.

Measurement	Males		Females	
	AI (n = 30)	HiD (n = 25)	AI (n = 24)	HiD (n = 21)
Food intake (g/d)*	3.4±0.1	3.2±0.1	3.3±0.1	3.0±0.1
Food intake (mg/g b.wt./d)*	154.8±2.8	153.2±3.0	188.7±4.1	172.6±4.4
Vitamin D_3_ intake (IU D_3_/d)	3.4±0.1	161.0±3.2	3.3±0.1	149.4±4.3
Vitamin D_3_ intake (IU D_3_/g b. wt./d)	0.155±0.003	7.661±0.151	0.189±0.004	8.631±0.218
Body weight (g)*	22.3±0.3	21.4±0.4	17.6±0.2	17.4±0.2

AI, adequate intake, n = 54; HiD, high vitamin D_3_, n = 46; b.wt: body weight.

* HiD females consumed 10% less absolute feed (P = 0.008) and 9% less feed corrected for body weight vs. AI females (P = 0.010). Males (154.0±2.4 mg/g b.wt./d) consumed 15% less food corrected for body weight vs. females (180.7±2.6 mg/g b.wt./d) (P<0.001). Males (21.9±0.2 g) had 25% higher body weight vs. females (17.5±0.2 g) (P<0.001). Data are means ± SEM.

### Body weight

Body weight was not significantly different between the diets (Table 2). AI mice had an average body weight of 20.2±0.4 g, and HiD mice had an average body weight of 19.6±0.4 g (Figure S1C).

### Body condition (BC)

BC was not significantly different between the diets (Table 3). Between age 60–141 d, males had an 11% lower BC vs. females (Table 4; Figure S1D). During disease progression, males had a 29% lower BC vs. females. Over time, BC was significantly lower than baseline starting at age 107 d (P≤0.001); starting at 107 d for males (P≤0.001) and at 113 d for females (P≤0.001). Within males, BC was significantly lower than baseline starting at 103 d for AI (P≤0.012) and 107 d for HiD (P≤0.042). Within females, BC was significantly lower than baseline starting at 117 d for AI (P≤0.001) and 117 d for HiD (P≤0.001).

**Table 3:** Body condition, functional outcomes and clinical score between the diet groups in G93A mice.

Measurement	Age 105 d–141 d			Age 60 d – 141 d		
	AI (n = 31)	HiD (n = 28)	P value	AI (n = 31)	HiD (n = 28)	P value
Body condition	1.8±0.1	2.0±0.1	NS	2.9±0.1	3.0±0.1	NS
Ability to move	2.3±0.2	2.4±0.2	NS	3.2±0.1	3.2±0.1	NS
Paw grip endurance (s)*	36±5	50±5	P = 0.042	101±4	113±4	P = 0.015
Motor performance (s)	9±1	9±1	NS	18±1	17±1	NS
Clinical score	3.4±0.1	3.4±0.1	NS	1.9±0.1	1.9±0.1	NS

AI, adequate intake, n = 31; HiD, high vitamin D_3_, n = 28.

* HiD mice had a 37% and 12% greater paw grip endurance between ages 105 d – 141 d and 60 d – 141 d, respectively, vs. AI. Data are means ± SEM.

**Table 4:** Body condition, functional outcomes and clinical score between the sexes in G93A mice.

Measurement	Age 105 d – 141 d			Age 60 d – 141 d		
	Males (n = 33)	Females (n = 26)	P value	Males (n = 33)	Females (n = 26)	P value
Body condition	1.6±0.1	2.3±0.1	P<0.001	2.8±0.1	3.2±0.1	P<0.001
Ability to move	2.1±0.1	2.6±0.2	P = 0.013	3.1±0.1	3.3±0.1	P = 0.013
Paw grip endurance (s)	36±5	52±6	P = 0.015	101±4	115±4	P = 0.007
Motor performance (s)	10±1	12±1	P = 0.071	17±1	18±1	NS
Clinical score	3.6±0.1	3.2±0.1	P = 0.011	2.0±0.1	1.7±0.1	P = 0.016

Males, n = 33; females, n = 26. Data are means ± SEM.

### Ability to move (ATM)

**Figure 1:**
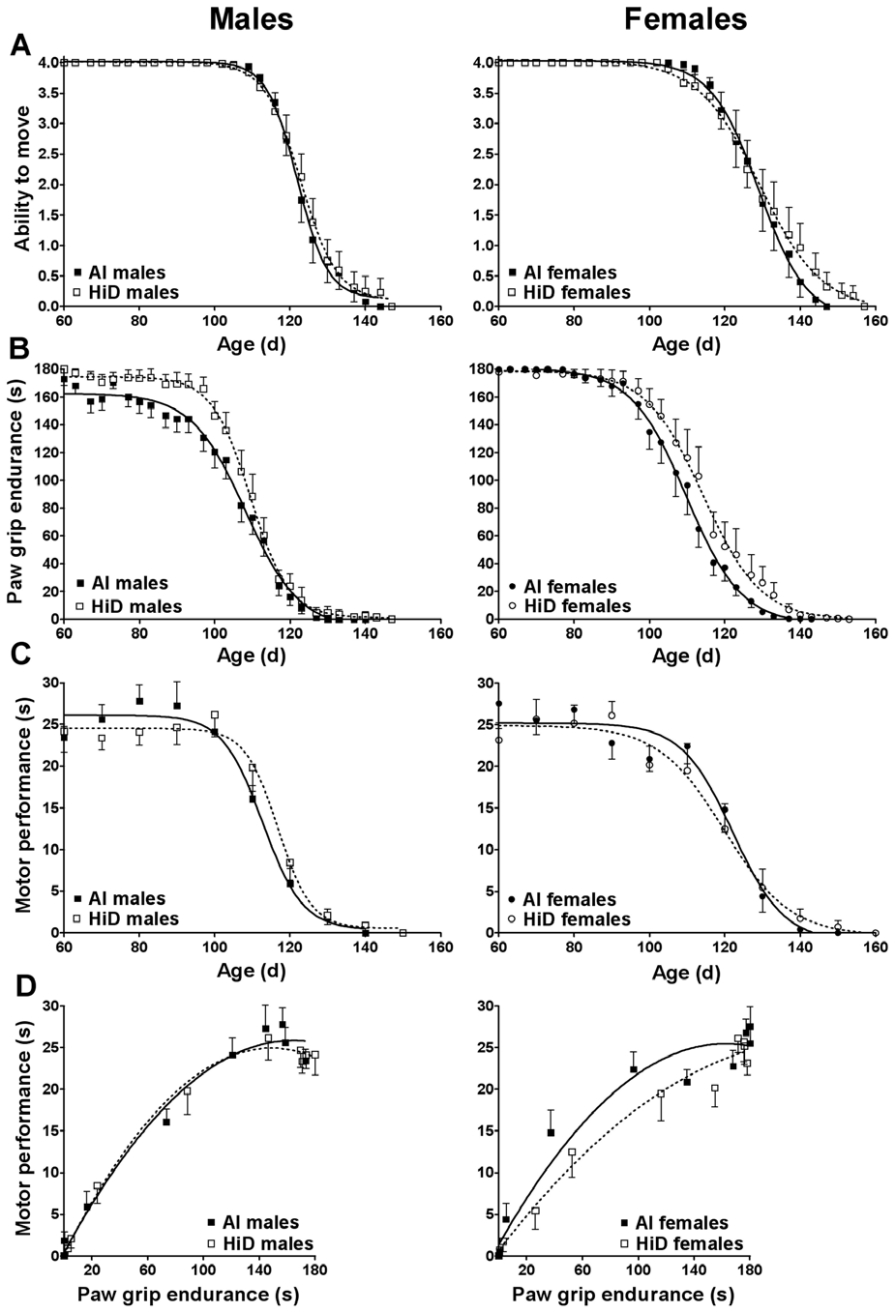
Functional outcomes over time and motor performance vs. paw grip endurance. A) Ability to move, B) paw grip endurance (s), C) motor performance (s) and D) correlation between motor performance (s) and paw grip endurance (s) for 31 adequate intake (AI; 1 IU D_3_/g feed; ▪, 18 males; •, 13 females) and 28 high (HiD; 50 IU D_3_/g feed; **□**, 15 males; **○**, 13 females) vitamin D_3_ G93A mice. A and C) There were no significant differences between the diets for ability to move or motor performance. B) For paw grip endurance, HiD mice had a 12% greater score between age 60–141 d (P = 0.015), a 9% greater score prior to disease onset (P = 0.020) and a 37% greater score during disease progression (P = 0.042) vs. AI mice. Differences were driven by a 13% greater score between age 60–141 d and prior to disease progression in HiD males (P = 0.032 and P = 0.024, respectively), and by a 43% greater score during disease progression in HiD females (P = 0.095). D) Corrected for paw grip endurance, HiD females had a 19% lower motor performance vs. AI females (P = 0.086). Data are means ± SEM.

ATM was not significantly different between the diets (Table 3). Between age 60–141 d, males had a 7% lower ATM vs. females (Table 4; Figure 1A). During disease progression, males had a 19% lower ATM vs. females. Over time, ATM was significantly lower than baseline starting at age 117 d (P≤0.001); starting at 117 d for males (P≤0.001) and at 120 d for females (P≤0.001). Within males, ATM was significantly lower than baseline starting at 117 d for AI (P≤0.003) and 117 d for HiD (P≤0.009). Within females, ATM was significantly lower than baseline starting at 120 d for AI (P≤0.019) and 123 d for HiD (P≤0.010).

### Paw grip endurance (PaGE)

Between age 60–141 d, HiD mice had a 12% greater PaGE vs. AI mice, which was driven by HiD males having a 13% greater PaGE vs. AI males (P = 0.032) (Table 3; Figure 1B). Prior to disease onset, HiD mice had a 9% greater PaGE vs. AI mice (P = 0.020), which was driven by HiD males having a 13% greater PaGE vs. AI males (P = 0.024). During disease progression, HiD females had a 43% greater PaGE vs. AI females (P = 0.095). Between the sexes, males had a 12% lower PaGE between age 60–141 d, a 10% lower PaGE prior to disease onset (P = 0.027), and a 32% lower PaGE during disease progression vs. females (Table 4). Over time, PaGE was significantly lower than baseline starting at age 97 d (P≤0.001); starting at 97 d for males (P≤0.001) and at 100 d for females (P≤0.001). Within males, PaGE was significantly lower than baseline starting at 97 d for AI (P≤0.001) and 100 d for HiD (P≤0.024). Within females, PaGE was significantly lower than baseline starting at 100 d for AI (P≤0.001) and 107 d for HiD (P≤0.004).

### Motor performance

MP was not significantly different between the diets or the sexes over time or for AUC (Table 3; Figure 1C). Over time, MP was significantly lower than baseline starting at age 110 d (P≤0.001); starting at 110 d for males (P≤0.001) and at 100 d for females (P≤0.025). Within males, MP was significantly lower than baseline starting at 110 d for AI (P≤0.001) and 120 d for HiD (P≤0.001). Within females, MP was significantly lower than baseline starting at 120 d for AI (P≤0.001) and 120 d for HiD (P≤0.001). When corrected for MP, HiD females trended toward a 19% lower PaGE AUC vs. AI females (P = 0.086) (Figure 1D).

### Clinical score (CS; disease severity)

**Figure 2:**
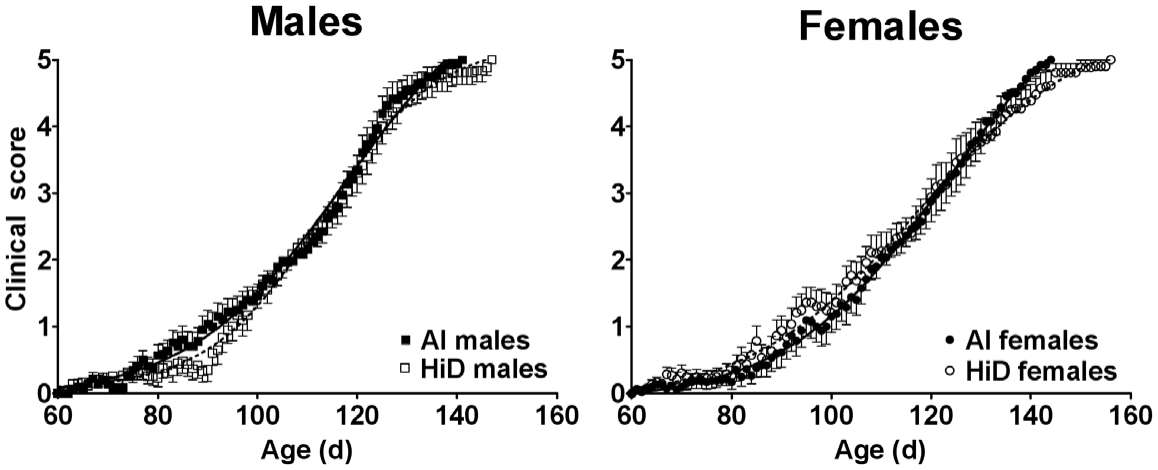
Clinical score over time. Clinical score for 31 adequate intake (AI; 1 IU D_3_/g feed; ▪, 18 males; •, 13 females) and 28 high (HiD; 50 IU D_3_/g feed; **□**, 15 males; **○**, 13 females) vitamin D_3_ G93A mice. Clinical score was not significantly different between the diets. Between the sexes, AI males had a 19% greater clinical score vs. AI females (P = 0.010), whereas there was no significant difference between HiD males and HiD females. Data are means ± SEM.

Prior to disease onset (age 60–105 d), HiD females had a non-significant 31% higher CS vs. AI females (Figure 2). In contrast, HiD males had a non-significant 19% lower CS vs. AI males prior to disease onset. Between the sexes, males had a 14% higher CS between age 60–141 d vs. females (Table 4); mainly driven by AI males having a 19% higher CS vs. AI females (P = 0.010). Prior to disease onset, AI males had a 44% higher CS vs. AI females (P = 0.058). During disease progression, males had a 14% higher CS vs. females (Table 4), mainly driven by AI males having a 14% higher CS vs. AI females (P = 0.026); whereas HiD males had a trend for an 8% higher CS vs. HiD females (P = 0.083). Over time, CS was significantly higher than baseline starting at age 81 d (P≤0.023); starting at 83 d for males (P≤0.043) and at 89 d for females (P≤0.012). Within males, CS was significantly higher than baseline starting at 88 d for AI (P≤0.021) and 94 d for HiD (P≤0.030). Within females, CS was significantly higher than baseline starting at 94 d for AI (P≤0.045) and 92 d for HiD (P≤0.018).

### BC, ATM, PaGE and MP vs. CS

BC, ATM and MP when corrected for CS, were not significantly different between the diets, although HiD males trended toward a 5% greater BC AUC vs. AI males (P = 0.065) (Figure S2A, S2B and S2D). However, corrected for CS, HiD mice had a 13% greater PaGE AUC vs. AI mice (P = 0.035), mainly driven by HiD females having a 20% greater AUC vs. AI females (P = 0.020) (Figure S2C). During disease progression, ATM and MP AUC negatively correlated with CS AUC for both AI (P<0.001; P = 0.035) and HiD (P<0.001; P = 0.087) mice, respectively (Figure S3A and S3B). Between 60 d-CS5, HiD mice had an 11% higher (P = 0.023) PaGE AUC corrected for CS AUC vs. AI mice (Figure S4A). Prior to disease onset, HiD mice had a 13% higher (P = 0.040) PaGE AUC corrected for CS AUC vs. AI mice. During disease progression, HiD mice had a 24% higher (P = 0.037) PaGE AUC corrected for CS AUC vs. AI mice (Figure S4B).

### Disease onset (CS2)

**Figure 3:**
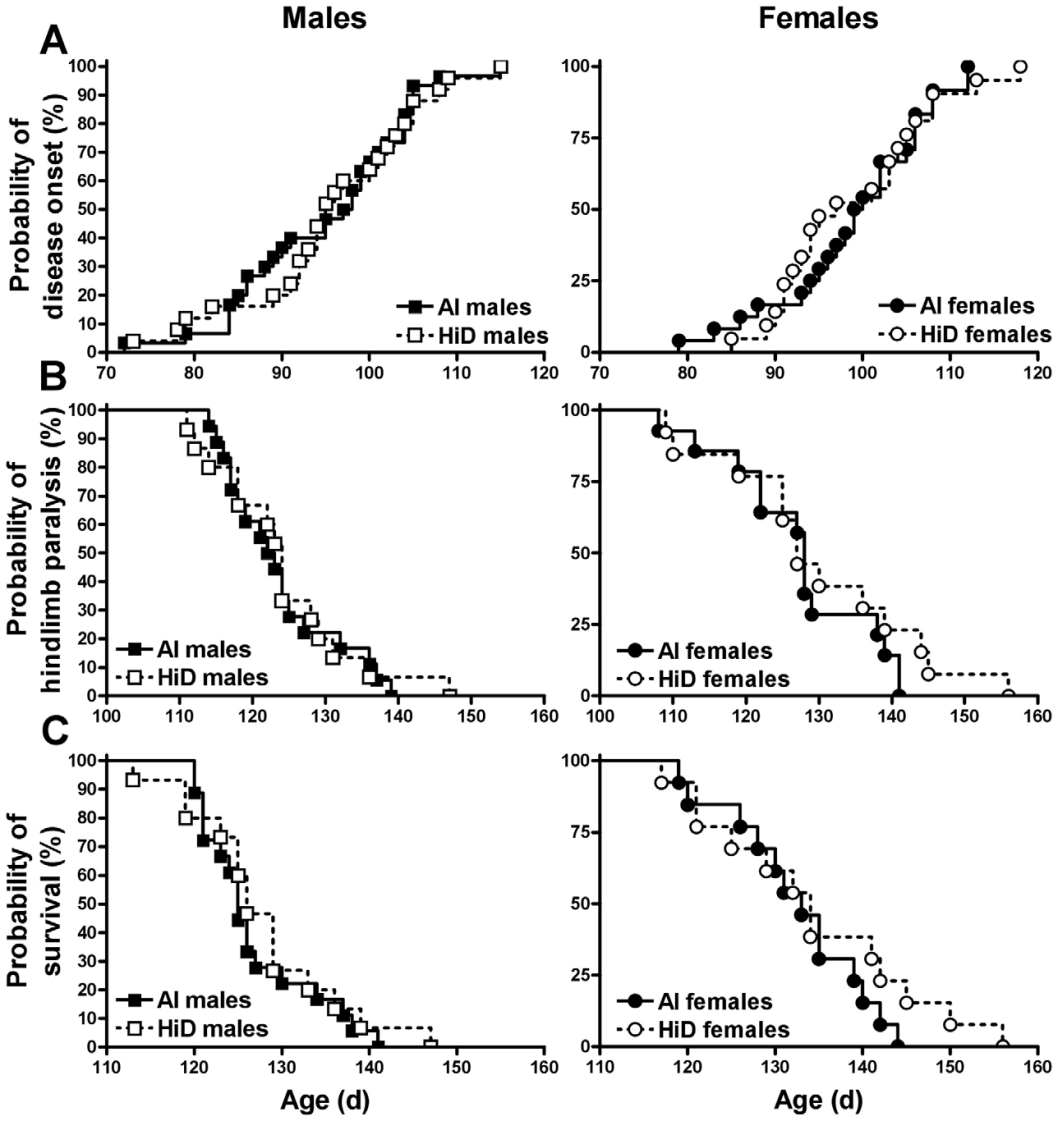
Probability of disease onset (CS2), hindlimb paralysis (CS4) and survival (CS5). A) Probability of CS2 for 54 adequate intake (AI; 1 IU D_3_/g feed; ▪, 30 males; •, 24 females) and 46 high (HiD; 50 IU D_3_/g feed; **□**, 25 males; **○**, 21 females) vitamin D_3_ G93A mice. B and C) Probability of CS4 and CS5 for 31 adequate intake (AI; 1 IU D_3_/g feed; ▪, 18 males; •, 13 females) and 28 high (HiD; 50 IU D_3_/g feed; **□**, 15 males; **○**, 13 females) vitamin D_3_ G93A mice. A) Males tended to reach CS2 at a 35% faster rate (i.e. the hazard ratio) vs. females (HR  = 1.35, 95% CI: 0.93, 2.14; P = 0.055). AI males tended to reach CS2 at a 40% faster rate vs. AI females (HR  = 1.40, 95% CI: 0.83, 2.67; P = 0.090), whereas there were no significant differences between HiD males and HiD females. B) Males reached CS4 at a 67% faster rate vs. females (HR  = 1.67, 95% CI: 1.05, 3.14; P = 0.016). AI males had a 78% faster rate of reaching CS4 vs. AI females (HR  = 1.78, 95% CI: 0.9, 4.3; P = 0.038). HiD males tended to reach CS4 at a 67% faster rate vs. HiD females (HR  = 1.67, 95% CI: 0.8, 4.0; P = 0.075). C) Males reached CS5 at an 89% faster rate vs. females (HR  = 1.89, 95% CI: 1.22, 3.76; P = 0.004). AI males had a 99% faster rate of reaching endpoint vs. AI females (HR  = 1.99, 95% CI: 1.05, 4.95; P = 0.018), whereas HiD males had an 81% faster rate vs. HiD females (HR  = 1.81, 95% CI; 0.91, 4.75; P = 0.042).

CS2 was not significantly different between the diets (Table 5; Figure 3A). Between the sexes, the age of mice at CS2 was 4% sooner for males vs. females (Table 6). The Logrank test revealed that males tended to reach CS2 at a 35% faster rate (i.e., the hazard ratio) vs. females (HR  = 1.35, 95% CI: 0.93, 2.14; P = 0.055). Within AI, males trended toward a 40% faster rate of reaching disease onset vs. females (HR  = 1.40, 95% CI: 0.83, 2.67; P = 0.090), whereas there were no differences between the sexes within the HiD diet.

**Table 5:** Disease outcomes and tissue weights between the diet groups in G93A mice.

Measurement	AI (n = 31)	HiD (n = 28)	P value
Age at disease onset (d)*	97±1	97±1	NS
Age at functional hindlimb paralysis (d)	125±2	127±2	NS
Age at endpoint (d)	129±1	131±2	NS
Disease progression (d)	29±2	31±2	NS
*Quadriceps* wt. (mg/g b.wt.) †	10.6±0.5	10.6±0.4	NS
*Tibialis anterior* wt. (mg/g b.wt.) †	3.02±0.13	3.15±0.10	NS
Brain wt. (mg/g b.wt.) †	19.4±0.5	19.6±0.5	NS

AI, adequate intake, n = 31; HiD, high vitamin D_3_, n = 28.

* AI, n = 54; HiD, n = 46.

† AI, n = 23; HiD, n = 18. Data are means ± SEM.

**Table 6:** Disease outcomes and tissue weight between the sexes in G93A mice.

Measurement	Males (n = 33)	Females (n = 26)	P value
Age at disease onset (d)*	95±1	99±1	P = 0.029
Age at functional hindlimb paralysis (d)	124±2	129±2	P = 0.036
Age at endpoint (d)	127±2	133±2	P = 0.006
Disease progression (d)	28±2	32±2	NS
*Quadriceps* wt. (mg/g b.wt.) †	10.5±0.4	10.7±0.5	NS
*Tibialis anterior* wt. (mg/g b.wt.) †	3.0±0.1	3.1±0.1	NS
Brain wt. (mg/g b.wt.) †	17.9±0.3	21.4±0.4	NS

Males, n = 33; females, n = 26.

* Males, n = 55; females, n = 45.

† Males, n = 22; females n = 19. Data are means ± SEM.

### Hindlimb paralysis (CS4) and survival (CS5)

Neither CS4 nor CS5 was significantly different between the diets (Table 5; Figure 3B, 3C). Between the sexes, the age of mice at CS4 was 4% sooner for males vs. females (Table 6). The Logrank test revealed that males reached CS4 at a 67% faster rate vs. females (HR  = 1.67, 95% CI: 1.05, 3.14; P = 0.016). Within AI, males had a 78% faster rate of reaching CS4 vs. females (HR  = 1.78, 95% CI: 0.9, 4.3; P = 0.038). Within HiD, males trended toward a 67% faster rate of reaching CS4 (HR  = 1.67, 95% CI: 0.8, 4.0; P = 0.075). Between the sexes, the age of mice at CS5 was 5% sooner for males vs. females (Table 6). The Logrank test revealed that males reached CS5 at an 89% faster rate vs. females (HR  = 1.89, 95% CI: 1.22, 3.76; P = 0.004). Within AI, males had a 99% faster rate of reaching endpoint vs. AI females (HR  = 1.99, 95% CI: 1.05, 4.95; P = 0.018), whereas HiD males had an 81% faster rate vs. HiD females (HR  = 1.81, 95% CI; 0.91, 4.75; P = 0.042).

### Disease progression

Disease progression was not significantly different between the diets or the sexes (Tables 5 and 6).

### *Tibialis anterior* weights

**Figure 4:**
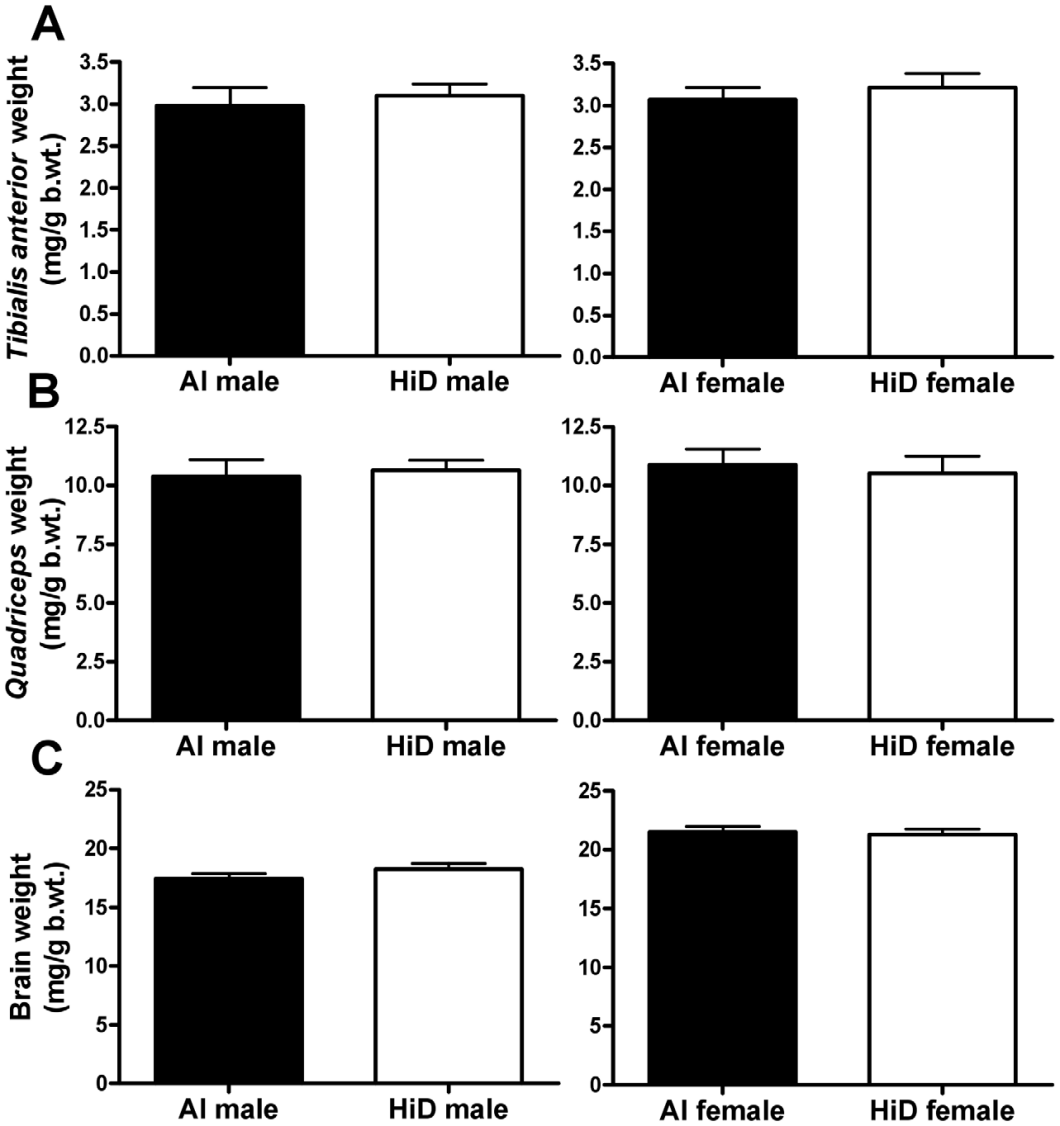
Body weight-adjusted *tibialis anterior*, *quadriceps* and brain weights. Body weight-adjusted A) *tibialis anterior* weight (mg/g b.wt.), B) *quadriceps* weight (mg/g b.wt.) and C) brain weight (mg/g b.wt.) for 23 adequate intake (AI; 1 IU D_3_/g feed; ▪, 12 males; •, 11 females) and 18 high (HiD; 50 IU D_3_/g feed; **□**, 10 males; **○**, 8 females) vitamin D_3_ G93A mice. There were no significant differences between the diets in body weight-adjusted *tibialis anterior, quadriceps* or brain weights. Data are means ± SEM.

Body weight-adjusted *tibialis anterior* weights were not significantly different between the diets or the sexes (Tables 5 and 6; Figure 4A). Between the sexes, males had 16% heavier absolute *tibialis anterior* vs. females (P = 0.010). Body weight-adjusted *tibialis anterior* weights positively correlated with age at CS2 for both AI (P<0.001) and HiD (P = 0.090) mice (Figure S5A).

### *Quadriceps* weights

Body weight-adjusted *quadriceps* weights were not significantly different between the diets or the sexes (Tables 5 and 6; Figure 4B). Between the sexes, males had 19% heavier absolute *quadriceps* vs. females (P = 0.011). Body weight-adjusted *quadriceps* weights positively correlated with age at CS2 for AI mice (P<0.001), but not for HiD mice (Figure S5B).

### Brain weights

Body weight-adjusted brain weights were not significantly different between the diets (Table 5; Figure 4C). Between the sexes, males had 17% lighter body weight-adjusted brain weights vs. females (Table 6). Body weight-adjusted brain weights positively correlated with age at CS4 (P = 0.014; Figure S6A) and CS5 (P = 0.018; Figure S6B).

## Discussion

The attenuated decline in paw grip endurance (PaGE) is supported by our previous study in the same G93A mouse model of ALS [27], as well as literature describing vitamin D and its relationship with muscle function in animals and humans. The presence of the vitamin D-receptor (VDR) in human skeletal muscle indicates a role for vitamin D in skeletal muscle function [18]. In support, VDR null mice are characterized by low body weight and impaired functional capacity [32]–[34]. VDR null mice have 20% smaller muscle fibre diameter as early as 21 d of age, worsening at 8 wk of age; a decrement that occurred pervasively without preference to muscle fibre type [35]. In addition, we have previously demonstrated that vitamin D_3_ restriction in G93A mice compounds the decrements observed in PaGE [36]. Further to animal studies, a significant body of literature in humans demonstrates improvements in functional capacity following vitamin D supplementation. In double-blind, randomized, placebo-controlled trials, supplementing 75–88 y old men and women (serum calcidiol <50 nmol/L) with 800 IU vitamin D_3_/d decreases the risk of falls by 27–72% [37]–[40].

Despite improvements in PaGE, vitamin D_3_ supplementation at 50 IU/g feed in the current study did not delay the age at disease onset and hindlimb paralysis, or prolong lifespan vs. AI mice, possibly due to tissue-specific effects of vitamin D in the CNS vs. skeletal muscle. A number of different interventions have demonstrated the effectiveness of calcitriol in the CNS [15], [41]–[46]. In rodent EAE, the beneficial effects of calcitriol are well established as disease can be fully prevented [41] or progression can be halted upon supplementation [41]. Underscoring its influence, subsequent withdrawal of calcitriol results in the resumption of disease progression [41]. These effects may be due to the marked reduction in brain macrophages and activated microglia as well as an almost complete inhibition of macrophage and microglia CD4 expression [42]. Also, calcitriol treatment greatly reduced EAE inducible nitric oxide synthase (iNOS) expression in the cerebellum, brain stem and spinal cord at the mRNA and protein levels [43]. Furthermore, calcitriol pretreatment reduced the volume of infarcted rat brain tissue induced by cerebral artery ligation by 2.3 fold, possibly due to the 2 fold increase in glial cell line-derived neurotrophic factor (GDNF) [14], and greatly attenuated the hypokinesia (reduction in voluntary movement) experienced by rats subjected to medial forebrain lesioning by up to 2 fold [47]. Indeed, even in healthy wild-type rats, calcitriol administration increased brain GDNF expression by 40% vs. saline-treated controls [15].

Alternatively, evidence suggests that calcidiol possesses functions in muscle tissue independent of calcitriol. *In vivo*, both a single oral dose of 400 IU vitamin D_3_ and intravenous injection of 0.4 µg calcidiol in vitamin D_3_-deficient rats significantly increased muscle leucine incorporation by 33% and 26% at 7 h and 4 h for vitamin D_3_ and calcidiol, respectively, compared to untreated controls [48]. Removal of the kidneys (and therefore the ability to renally convert calcidiol to calcitriol) did not abolish the effect of vitamin D_3_ administration, demonstrating a direct role of calcidiol independent of calcitriol in muscle function [48]. In evidence, *in vitro* rat epitrochlear muscle had greater leucine incorporation and ATP content in a medium containing 50 nmol/L calcidiol, but not 52,000 nmol/L vitamin D_3_ or 1.2 nmol/L calcitriol, vs. untreated muscle [48], indicating that vitamin D_3_'s action in skeletal muscle is conditional upon its conversion to calcidiol, and that calcidiol is the active vitamin D metabolite in skeletal muscle. Indeed, vitamin D_3_ supplementation rescues growth retardation observed in CYP27B1 knockout mice (CYP27B1 is the enzyme that converts calcidiol to calcitriol) [49], [50]. Thus, we theorize that vitamin D metabolites, specifically calcidiol, may have exacted improvements in muscle function due to optimal intramyocellular vitamin D status, whereas calcitriol could not rescue the neurodegeneration characteristic of this disease model.

In the current study, females consuming the HiD diet (50 IU/g feed) may have reached vitamin D_3_ toxicity as indicated by a significantly reduced food intake (absolute and corrected for body weight) and 31% greater disease severity prior to disease onset. Their vitamin D_3_ intake is equivalent to 40,582 IU/d for an 80 kg man and 39,049 IU/d for a 70 kg woman. The observed signs of toxicity in HiD females could be explained by sex differences in vitamin D metabolism. In B10.PL mice supplemented orally with 40 IU vitamin D_3_/d, females exhibited a ∼40% and ∼37% greater serum calcidiol at 38 d and 83 d vs. supplemented males, respectively, despite similar baseline serum calcidiol [21]. In the spinal cord, females had ∼67% greater calcitriol concentrations vs. males 70–84 d post-supplementation despite similar serum values. In line with this, supplemented females also exhibited a ∼2 fold and ∼4 fold lower kidney and spinal cord CYP24A1 (the calcitriol deactivating enzyme) mRNA transcripts, respectively, vs. supplemented males [21]. Nashold et al. propose a mechanism for observed synergy between vitamin D_3_ and estrogen: estrogen enhances vitamin D function by increasing net calcitriol concentration via estrogen receptor-mediated down-regulation of CYP24A1 transcription. Estrogen increases vitamin D potency via the up-regulation of VDR, and, in turn, calcitriol enhances endogenous estrogen synthesis via VDR-mediated up-regulation of estrogen synthase [51].

Taken together, the above data could explain the apparent paradox observed in the HiD G93A females in the current study: despite mild signs of vitamin D_3_ toxicity, HiD females had greater PaGE compared to their AI female counterparts. Vitamin D_3_ toxicity may have been a result of high CNS calcitriol. In limited evidence, female mice consuming 40 IU vitamin D_3_/d (∼3.0 IU/g b.wt./d) had spinal cord calcitriol of ∼125 fmol/g [21]. This is equivalent to 2.5 fold higher than vitamin D deficient females which had concentrations considered to be above normal given that CYP27B1 expression is increased whereas that for CYP24A1 is decreased under vitamin D deficiency [52]–[54]. In the current study, HiD females consumed ∼150 IU vitamin D_3_/d (8.6 IU/g b.wt./d) likely resulting in supraphysiological levels of spinal cord calcitriol concentrations, inducing mild toxicity as evidenced by greater disease severity prior to disease onset and decreased food intake. However, considering that skeletal muscle is unable to perform the calcidiol-to-calcitriol conversion [48], muscle tissue, but not the CNS, may have been spared the sequelae associated with hypercalcitriol-induced toxicity in HiD G93A females.

In closing, the current study confirms our previous observation that dietary vitamin D_3_ supplementation above the AI attenuates the decline in PaGE, but does not delay the age at disease onset, hindlimb paralysis or endpoint in the G93A mouse model of ALS.

## Supporting Information

Figure S1 **Food intake, food intake corrected for body weight, body weight and body condition over time.** A) Food intake (g), B) food intake corrected for body weight (mg/g b.wt.), C) body weight (g) for 54 adequate intake (AI; 1 IU D_3_/g feed; ▪, 30 males; •, 24 females) and 46 high (HiD; 50 IU D_3_/g feed; **□**, 25 males; **○**, 21 females) vitamin D_3_ G93A mice, and D) body condition for 31 adequate intake (AI; 1 IU D_3_/g feed; ▪, 18 males; •, 13 females) and 28 high (HiD; 50 IU D_3_/g feed; **□**, 15 males; **○**, 13 females) vitamin D_3_ G93A mice. A and B) HiD females consumed 10% less food (P = 0.008) and 9% less food corrected for body weight (P = 0.010) vs. AI females. C and D) There were no significant differences between the diets for body weight or body condition. Data are means ± SEM.
(TIF)

Figure S2 **Relationship between functional outcomes and clinical score.** A) Body condition vs. clinical score, B) ability to move vs. clinical score, C) paw grip endurance vs. clinical score and D) motor performance vs. clinical score for 31 adequate intake (AI; 1 IU D_3_/g feed; ▪, 18 males; •, 13 females) and 28 high (HiD; 50 IU D_3_/g feed; **□**, 15 males; **○**, 13 females) vitamin D_3_ G93A mice. A) Corrected for clinical score, HiD males had a 5% greater body condition AUC vs. AI males (P = 0.065). B and D) Ability to move and motor performance vs. clinical score were not significantly different between the diets. C) Corrected for clinical score, HiD mice had a 13% greater paw grip endurance AUC vs. AI mice, mainly driven by a 20% greater AUC in HiD females vs. AI females. Data are means ± SEM.
(TIFF)

Figure S3 **Relation between ability to move AUC and motor performance AUC vs. clinical score AUC.** A) Ability to move AUC and B) motor performance AUC vs. clinical score AUC between CS2 – CS5 (during disease progression) for 31 adequate intake (AI; 1 IU D_3_/g feed; ▪, 18 males; •, 13 females) and 28 high (HiD; 50 IU D_3_/g feed; **□**, 15 males; **○**, 13 females) vitamin D_3_ G93A mice. A) During disease progression, ability to move AUC negatively correlated with CS AUC for both AI (r = −0.965; slope  = −1.17; P<0.001) and HiD (r = −0.949; slope  = −1.02 P<0.001) mice. For AI mice: ATM AUC_CS2 – CS5_  =  (6.452±0.16) + [(−1.17±0.06) × (CS AUC_CS2 – CS5_)]. For HiD mice: ATM AUC_CS2 – CS5_  =  (6.06±0.18) + [(−1.02±0.07) × (CS AUC_CS2 – CS5_)]. B) During disease progression, motor performance AUC negatively correlated with CS AUC for both AI (r = −0.380; slope  = −7.61; P = 0.035) and HiD (r = −0.330; slope  = −7.46; P = 0.087) mice. For AI mice: MP AUC_CS2 – CS5_  =  (34.52±9.14) + [(−7.61±3.44) × (CS AUC_CS2 – CS5_)]. For HiD: MP AUC_CS2 – CS5_  =  (34.24±11.34) + [(−7.46±4.19) × (CS AUC_CS2 – CS5_)].
(TIFF)

Figure S4 **Relation between paw grip endurance and clinical score area under the curve (AUC).** A) Paw grip endurance AUC vs. clinical score AUC between age 60 d – CS5 for 18 adequate intake (AI; 1 IU D_3_/g feed; ▪) and 15 high (HiD; 50 IU D_3_/g feed; **□**) vitamin D_3_ G93A male mice. B) Paw grip endurance AUC vs. clinical score AUC between CS2 – CS5 (during disease progression) for 13 adequate intake (AI; 1 IU D_3_/g feed; •) and 13 high (HiD; 50 IU D_3_/g feed; **○**) vitamin D_3_ G93A female mice. A) Between age 60 d – CS5, HiD males (r = 0.001; slope  = −0.10; P = 0.997) had an 18% greater PaGE AUC elevation (P = 0.082) vs. AI males (r = 0.087; slope  = −8.33; P = 0.731). For AI males: PaGE AUC_60 d – CS5_  =  (126.4±33.64) + [(−8.33±23.84)) × (CS AUC_60 d – CS5_)]. For HiD males: PaGE AUC_60 d – CS5_  =  (128.6±30.68) + [(−0.1043±23.35)) × (CS AUC_60 d – CS5_)]. B) During disease progression, HiD females (r = 0.260; slope  = −30.99; P = 0.391) had a 59% greater PaGE AUC elevation (P = 0.008) vs. AI females (r = 0.166; slope  = −14.98; P = 0.588). For AI females: PaGE AUC_CS2 – CS5_  =  (88.79±72.00) + [(−14.98±26.84) × (CS AUC_CS2 – CS5_)]. For HiD females: PaGE AUC_CS2 – CS5_  =  (160.1±93.10) + [(−30.99±34.72) × (CS AUC_CS2 – CS5_)].
(TIFF)

Figure S5 **Relation between body weight-adjusted** ***tibialis anterior*** **and** ***quadriceps*** **vs. disease onset (CS2).** A) *Tibialis anterior* weight (mg/g b.wt.) vs. age at CS2 and B) *quadriceps* weight (mg/g b.wt.) vs. age at CS2 for 23 adequate intake (AI; 1 IU D_3_/g feed; ▪, 12 males; •, 11 females) and 18 high (HiD; 50 IU D_3_/g feed; **□**, 10 males; **○**, 8 females) vitamin D_3_ G93A mice. A) *Tibialis anterior* weight (mg/g b.wt) positively correlated with age at CS2 for both AI (r = 0.662; slope  = 10.05; P<0.001) and HiD (r = 0.411; slope  = 8.482; P = 0.090) mice. For AI mice: age at CS2 (d)  =  (62.47±7.67) + [(10.05±2.49) × (*tibialis anterior* weight (mg/g b.wt.))]. For HiD mice: age at CS2 (d)  =  (65.68±14.94) + [(8.48±4.70) × (*tibialis anterior* weight (mg/g b.wt.))]. B) *Quadriceps* weight (mg/g b.wt.) positively correlated with age at CS2 for AI (r = 0.661; slope  = 2.735 P<0.001) but not for HiD (r = 0.229; slope  = 1.27; P = 0.361) mice. For AI mice: age at CS2 (d)  =  (63.74±7.36) + [(2.74±0.68) × (*quadriceps* weight (mg/g b.wt.))]. For HiD mice: age at CS2 (d)  =  (78.90±14.51) + [(1.27±1.35) × (*quadriceps* weight (mg/g b.wt.))].
(TIFF)

Figure S6 **Group body weight-adjusted brain weights vs. group age at hindlimb paralysis (CS4) and endpoint (CS5).** Group mean brain weights (mg/g b.wt.) for 23 adequate intake (AI; 1 IU D_3_/g feed; ▪, 12 males; •, 11 females) and 18 high (HiD; 50 IU D_3_/g feed; **□**, 10 males; **○**, 8 females) vitamin D_3_ G93A mice vs. mean group age at A) CS4 and B) CS5 for 31 adequate intake (AI; 1 IU D_3_/g feed; 18 males; 13 females) and 28 high (HiD; 50 IU D_3_/g feed; 15 males; 13 females) vitamin D_3_ G93A mice. A) Body weight-adjusted brain weights positively correlated with age at CS4 (r = 0.986; slope  = 1.64; P = 0.014). Age at CS4 (d)  =  (94.55±3.90) + [(1.64±0.199) × (brain weights (mg/g b.wt.))]. B) A) Body weight-adjusted brain weights positively correlated with age at CS5 (r = 0.982; slope  = 1.79; P = 0.018). Age at CS5 (d)  =  (95.47±4.75) + [(1.79±0.24) × (brain weights (mg/g b.wt.))]. Data are means ± SEM.
(TIFF)

Results S1 (DOC)
