# Supplementary figures and images for: Vitamin D3 at 50x AI Attenuates the Decline in Paw Grip Endurance, but Not Disease Outcomes, in the G93A Mouse Model of ALS, and Is Toxic in Females
Source: PLoS One. 2013 Feb 6;8(2):e30243. doi: 10.1371/journal.pone.0030243 (PMC3566148; doi:10.1371/journal.pone.0030243)

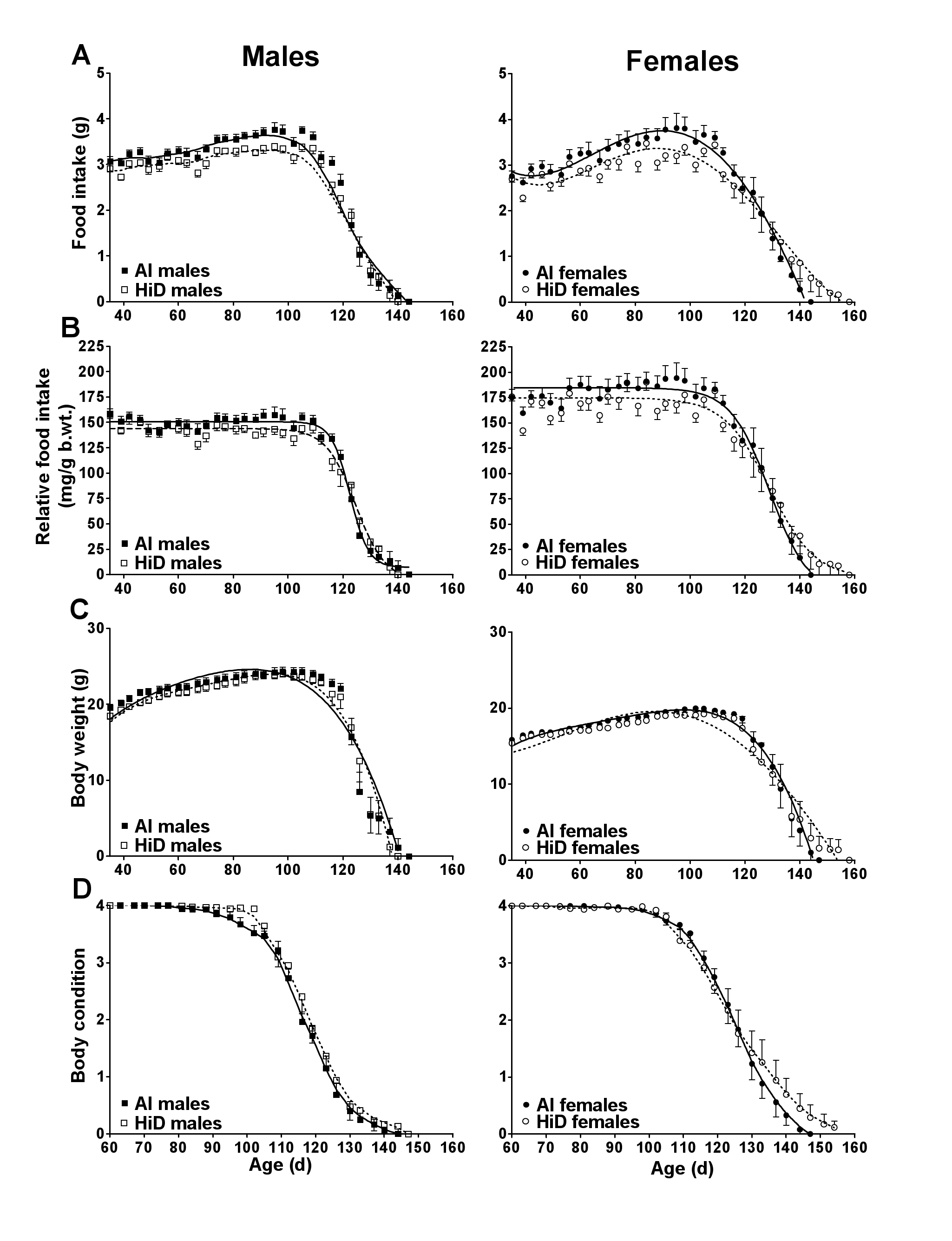

Supplement: Figure S1 — Food intake, food intake corrected for body weight, body weight and body condition over time. A) Food intake (g), B) food intake corrected for body weight (mg/g b.wt.), C) body weight (g) for 54 adequate intake (AI; 1 IU D3/g feed; ▪, 30 males; •, 24 females) and 46 high (HiD; 50 IU D3/g feed; □, 25 males; ○, 21 females) vitamin D3 G93A mice, and D) body condition for 31 adequate intake (AI; 1 IU D3/g feed; ▪, 18 males; •, 13 females) and 28 high (HiD; 50 IU D3/g feed; □, 15 males; ○, 13 females) vitamin D3 G93A mice. A and B) HiD females consumed 10% less food (P = 0.008) and 9% less food corrected for body weight (P = 0.010) vs. AI females. C and D) There were no significant differences between the diets for body weight or body condition. Data are means ± SEM. (TIF) [file pone.0030243.s001.tif]

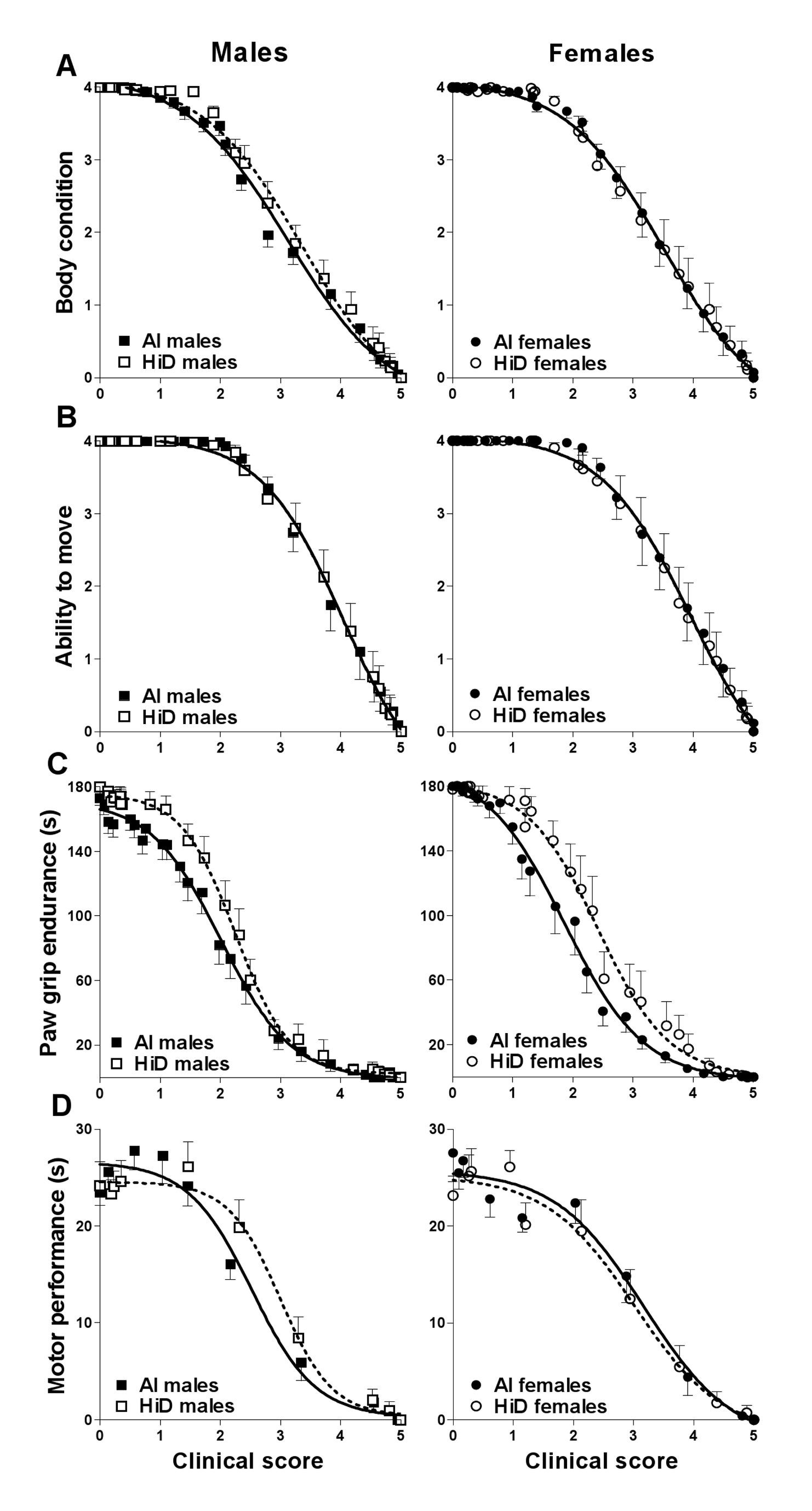

Supplement: Figure S2 — Relationship between functional outcomes and clinical score. A) Body condition vs. clinical score, B) ability to move vs. clinical score, C) paw grip endurance vs. clinical score and D) motor performance vs. clinical score for 31 adequate intake (AI; 1 IU D3/g feed; ▪, 18 males; •, 13 females) and 28 high (HiD; 50 IU D3/g feed; □, 15 males; ○, 13 females) vitamin D3 G93A mice. A) Corrected for clinical score, HiD males had a 5% greater body condition AUC vs. AI males (P = 0.065). B and D) Ability to move and motor performance vs. clinical score were not significantly different between the diets. C) Corrected for clinical score, HiD mice had a 13% greater paw grip endurance AUC vs. AI mice, mainly driven by a 20% greater AUC in HiD females vs. AI females. Data are means ± SEM. (TIFF) [file pone.0030243.s002.tiff]

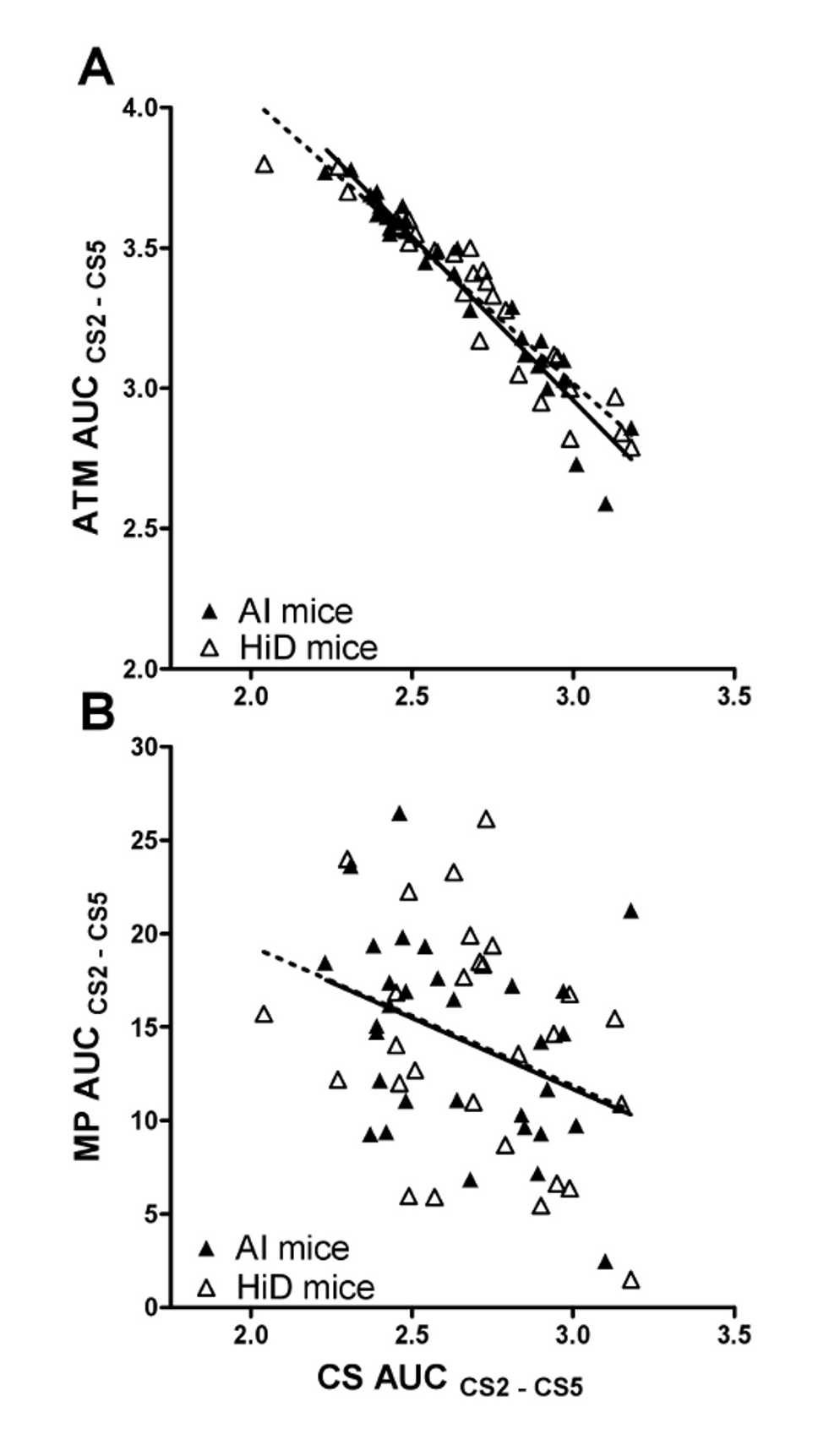

Supplement: Figure S3 — Relation between ability to move AUC and motor performance AUC vs. clinical score AUC. A) Ability to move AUC and B) motor performance AUC vs. clinical score AUC between CS2 – CS5 (during disease progression) for 31 adequate intake (AI; 1 IU D3/g feed; ▪, 18 males; •, 13 females) and 28 high (HiD; 50 IU D3/g feed; □, 15 males; ○, 13 females) vitamin D3 G93A mice. A) During disease progression, ability to move AUC negatively correlated with CS AUC for both AI (r = −0.965; slope = −1.17; P<0.001) and HiD (r = −0.949; slope = −1.02 P<0.001) mice. For AI mice: ATM AUCCS2 – CS5 = (6.452±0.16) + [(−1.17±0.06) × (CS AUCCS2 – CS5)]. For HiD mice: ATM AUCCS2 – CS5 = (6.06±0.18) + [(−1.02±0.07) × (CS AUCCS2 – CS5)]. B) During disease progression, motor performance AUC negatively correlated with CS AUC for both AI (r = −0.380; slope = −7.61; P = 0.035) and HiD (r = −0.330; slope = −7.46; P = 0.087) mice. For AI mice: MP AUCCS2 – CS5 = (34.52±9.14) + [(−7.61±3.44) × (CS AUCCS2 – CS5)]. For HiD: MP AUCCS2 – CS5 = (34.24±11.34) + [(−7.46±4.19) × (CS AUCCS2 – CS5)]. (TIFF) [file pone.0030243.s003.tiff]

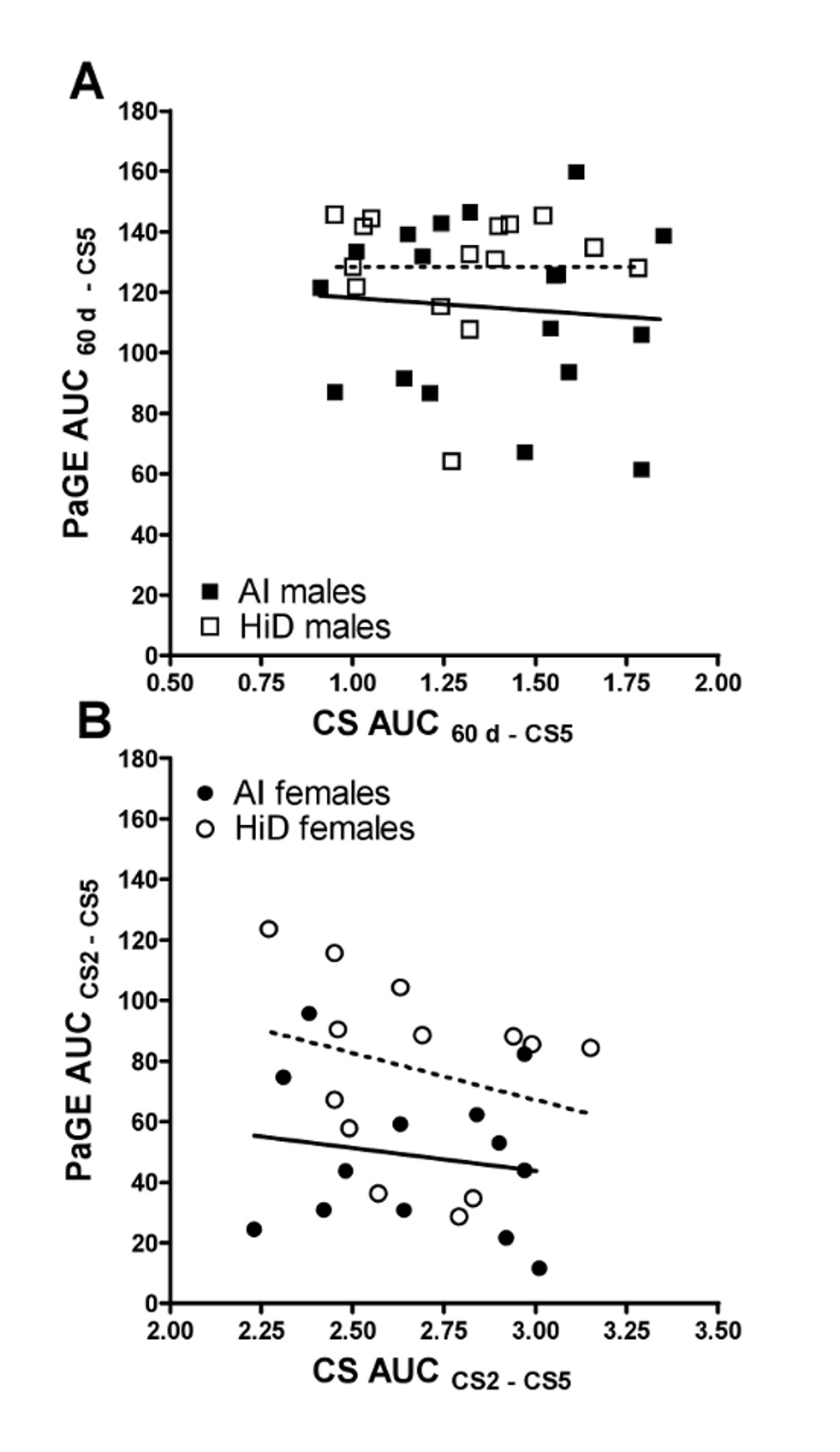

Supplement: Figure S4 — Relation between paw grip endurance and clinical score area under the curve (AUC). A) Paw grip endurance AUC vs. clinical score AUC between age 60 d – CS5 for 18 adequate intake (AI; 1 IU D3/g feed; ▪) and 15 high (HiD; 50 IU D3/g feed; □) vitamin D3 G93A male mice. B) Paw grip endurance AUC vs. clinical score AUC between CS2 – CS5 (during disease progression) for 13 adequate intake (AI; 1 IU D3/g feed; •) and 13 high (HiD; 50 IU D3/g feed; ○) vitamin D3 G93A female mice. A) Between age 60 d – CS5, HiD males (r = 0.001; slope = −0.10; P = 0.997) had an 18% greater PaGE AUC elevation (P = 0.082) vs. AI males (r = 0.087; slope = −8.33; P = 0.731). For AI males: PaGE AUC60 d – CS5 = (126.4±33.64) + [(−8.33±23.84)) × (CS AUC60 d – CS5)]. For HiD males: PaGE AUC60 d – CS5 = (128.6±30.68) + [(−0.1043±23.35)) × (CS AUC60 d – CS5)]. B) During disease progression, HiD females (r = 0.260; slope = −30.99; P = 0.391) had a 59% greater PaGE AUC elevation (P = 0.008) vs. AI females (r = 0.166; slope = −14.98; P = 0.588). For AI females: PaGE AUCCS2 – CS5 = (88.79±72.00) + [(−14.98±26.84) × (CS AUCCS2 – CS5)]. For HiD females: PaGE AUCCS2 – CS5 = (160.1±93.10) + [(−30.99±34.72) × (CS AUCCS2 – CS5)]. (TIFF) [file pone.0030243.s004.tiff]

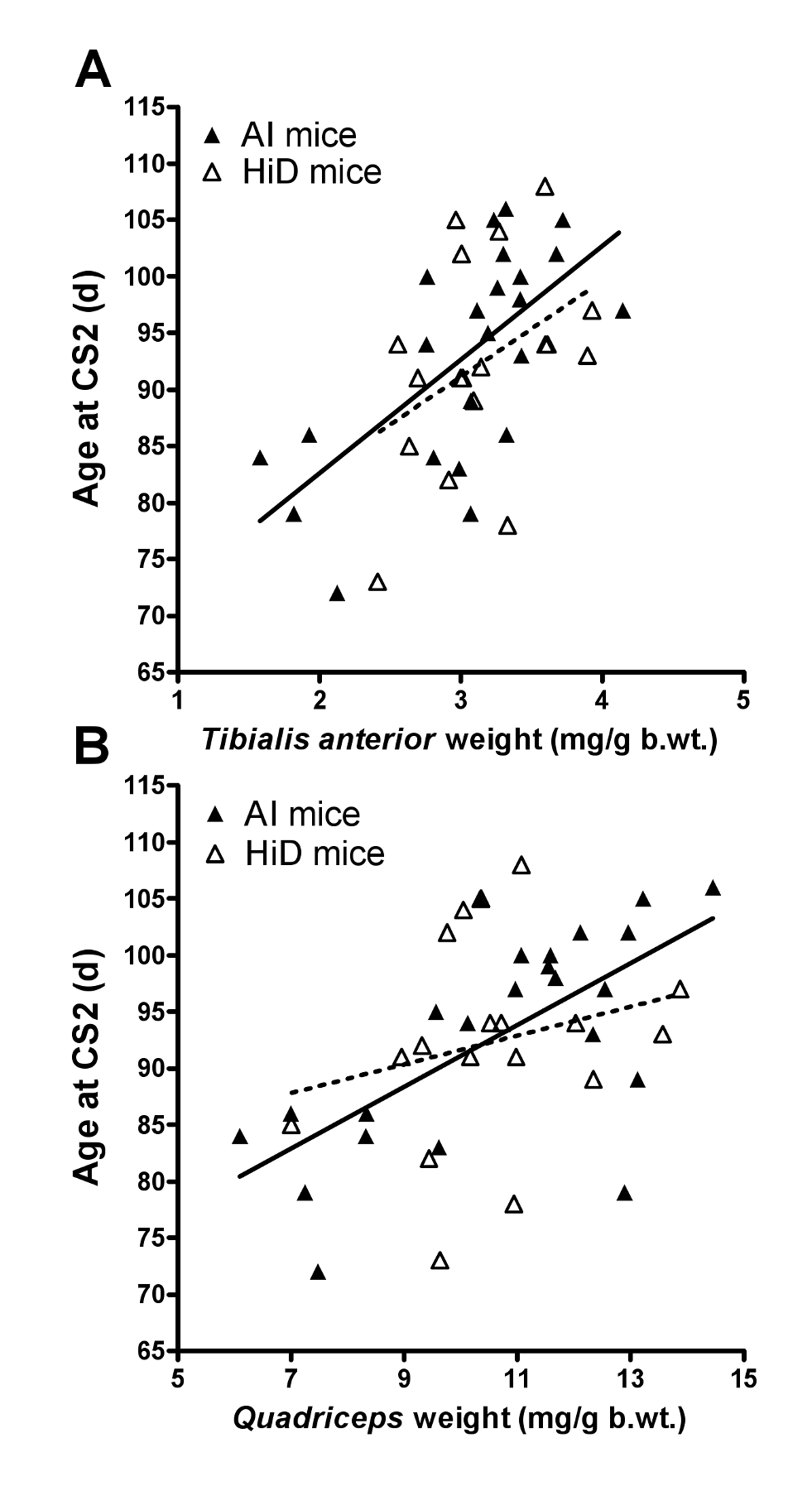

Supplement: Figure S5 — Relation between body weight-adjusted tibialis anterior and quadriceps vs. disease onset (CS2). A) Tibialis anterior weight (mg/g b.wt.) vs. age at CS2 and B) quadriceps weight (mg/g b.wt.) vs. age at CS2 for 23 adequate intake (AI; 1 IU D3/g feed; ▪, 12 males; •, 11 females) and 18 high (HiD; 50 IU D3/g feed; □, 10 males; ○, 8 females) vitamin D3 G93A mice. A) Tibialis anterior weight (mg/g b.wt) positively correlated with age at CS2 for both AI (r = 0.662; slope = 10.05; P<0.001) and HiD (r = 0.411; slope = 8.482; P = 0.090) mice. For AI mice: age at CS2 (d) = (62.47±7.67) + [(10.05±2.49) × (tibialis anterior weight (mg/g b.wt.))]. For HiD mice: age at CS2 (d) = (65.68±14.94) + [(8.48±4.70) × (tibialis anterior weight (mg/g b.wt.))]. B) Quadriceps weight (mg/g b.wt.) positively correlated with age at CS2 for AI (r = 0.661; slope = 2.735 P<0.001) but not for HiD (r = 0.229; slope = 1.27; P = 0.361) mice. For AI mice: age at CS2 (d) = (63.74±7.36) + [(2.74±0.68) × (quadriceps weight (mg/g b.wt.))]. For HiD mice: age at CS2 (d) = (78.90±14.51) + [(1.27±1.35) × (quadriceps weight (mg/g b.wt.))]. (TIFF) [file pone.0030243.s005.tiff]

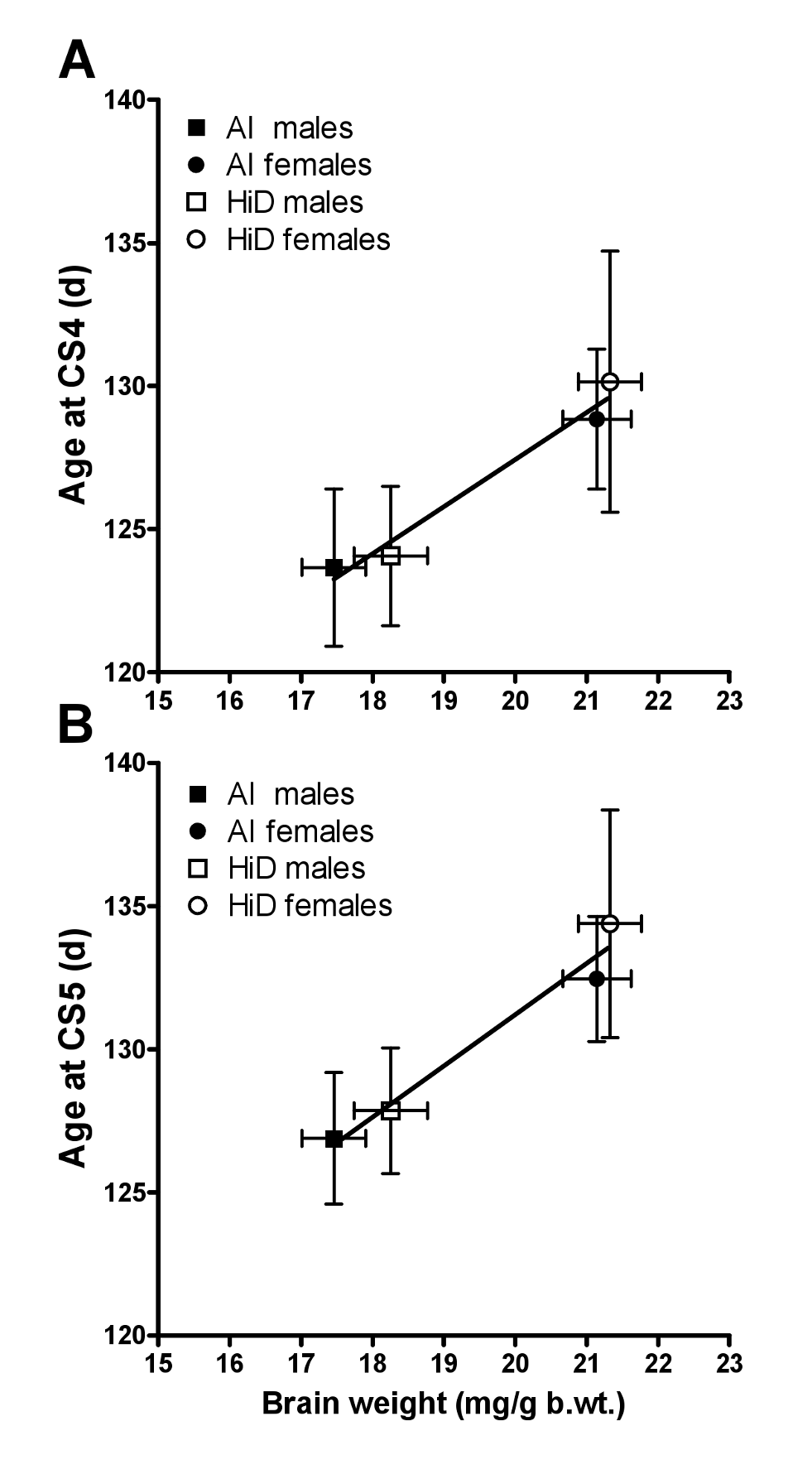

Supplement: Figure S6 — Group body weight-adjusted brain weights vs. group age at hindlimb paralysis (CS4) and endpoint (CS5). Group mean brain weights (mg/g b.wt.) for 23 adequate intake (AI; 1 IU D3/g feed; ▪, 12 males; •, 11 females) and 18 high (HiD; 50 IU D3/g feed; □, 10 males; ○, 8 females) vitamin D3 G93A mice vs. mean group age at A) CS4 and B) CS5 for 31 adequate intake (AI; 1 IU D3/g feed; 18 males; 13 females) and 28 high (HiD; 50 IU D3/g feed; 15 males; 13 females) vitamin D3 G93A mice. A) Body weight-adjusted brain weights positively correlated with age at CS4 (r = 0.986; slope = 1.64; P = 0.014). Age at CS4 (d) = (94.55±3.90) + [(1.64±0.199) × (brain weights (mg/g b.wt.))]. B) A) Body weight-adjusted brain weights positively correlated with age at CS5 (r = 0.982; slope = 1.79; P = 0.018). Age at CS5 (d) = (95.47±4.75) + [(1.79±0.24) × (brain weights (mg/g b.wt.))]. Data are means ± SEM. (TIFF) [file pone.0030243.s006.tiff]
