# Supplementary material for: Vitamin D3 at 50x AI Attenuates the Decline in Paw Grip Endurance, but Not Disease Outcomes, in the G93A Mouse Model of ALS, and Is Toxic in Females
Source: PLoS One. 2013 Feb 6;8(2):e30243. doi: 10.1371/journal.pone.0030243 (PMC3566148; doi:10.1371/journal.pone.0030243)
Supplement: Results S1 — (DOC) [file pone.0030243.s007.doc]

**SUPPLEMENTAL RESULTS**

*PaGE*

During disease progression, PaGE AUC positively correlated with BC AUC for all (r = 0.362, P = 0.005) and HiD (r = 0.519, P = 0.005) mice; HiD females had a 53% greater PaGE elevation vs. AI females (P = 0.026).

*Correlational analyses*

*BC vs. CS:* Corrected for CS, BC was not significantly different between the diets, although HiD males trended toward a 5% greater AUC vs. AI males (P = 0.065) (Figure 2A). Between the sexes, males had a 7% lower AUC vs. females (P = 0.001). BC was significantly lower than baseline starting at CS 2.25 (P ≤ 0.001); for both males and females. Within both sexes, BC was significantly lower than baseline starting at CS 2.25 for both AI and HiD (P ≤ 0.001). Data followed a sigmoidal relationship for males (AI males, r^2^ = 0.996; HiD males, r^2^ = 0.997; curves were significantly different, P = 0.016) and females (AI females, r^2^ = 0. 999; HiD females, r^2^ = 0.996; curves were not significantly different).

*ATM vs. CS:* Corrected for CS, ATM was not significantly different between the diets or the sexes (Figure 2B). ATM was significantly lower than baseline starting at CS 2.25 (P ≤ 0.001); starting at CS 2.25 for males (P ≤ 0.024) and at CS 2.5 for females (P ≤ 0.001). Within both sexes, ATM was significantly lower than baseline starting at CS 2.5 for both AI and HiD (P ≤ 0.001). Data followed a sigmoidal relationship for males (AI males, r^2^ = 0.998; HiD males, r^2^ = 0.999; curves were not significantly different) and females (AI females, r^2^ = 0.997; HiD females, r^2^ = 0.999; curves were not significantly different). Between 60 d-CS5, ATM AUC negatively correlated with CS AUC for all (r = -0.3148, P = 0.015) and HiD (r = -0.464, P = 0.013) mice, but not for AI mice. During disease progression, ATM AUC negatively correlated with CS AUC for both AI (r = -0.965, P < 0.001) and HiD (r = -0.949, P < 0.001) mice (Figure 3A).

*PaGE vs. CS:* Corrected for CS, HiD mice had a 13% greater PaGE AUC vs. AI mice (P = 0.035), mainly driven by HiD females having a 20% greater AUC vs. AI females (P = 0.020) (Figure 2C). PaGE was significantly lower than baseline starting at CS 0.75 (P ≤ 0.033); starting at CS 1.0 for males (P ≤ 0.014) and at CS 1.5 for females (P ≤ 0.001). Within males, PaGE was significantly lower than baseline starting at CS 1.5 for AI (P ≤ 0.003) and CS 2.0 for HiD (P ≤ 0.028). Within females, PaGE was significantly lower than baseline starting at CS 1.5 for AI (P ≤ 0.010) and CS 2.25 for HiD (P ≤ 0.001), with PaGE declining at a significantly later CS in HiD vs. AI females (CS x diet interaction P = 0.039). Data followed a sigmoidal relationship for males (AI males, r^2^ = 0.998; HiD males, r^2^ = 0.998; curves were significantly different, P < 0.001) and females (AI females, r^2^ = 0.996; HiD females, r^2^ = 0.993; curves were significantly different, P < 0.001). Between 60 d-CS5, HiD mice had an 11% higher (P = 0.023) PaGE AUC corrected for CS AUC vs. AI mice, mainly driven by HiD males having an 18% greater elevation vs. AI males (P = 0.082) (Figure 4A). Prior to disease onset, HiD mice had a 13% higher (P = 0.040) PaGE AUC corrected for CS AUC vs. AI mice, mainly driven by HiD males having a 19% greater elevation vs. AI males (P = 0.053). During disease progression, HiD mice had a 24% higher (P = 0.037) PaGE AUC corrected for CS AUC vs. AI mice, mainly driven by HiD females having a 59% greater (P = 0.008) elevation vs. AI females (Figure 4B).

*MP vs. CS* Corrected for CS, MP was not significantly different between the diets or the sexes (Figure 2D). MP was significantly lower than baseline starting at CS 1.0 (P ≤ 0.046); starting at CS 1.5 for males (P ≤ 0.018) and at CS 1.5 for females (P ≤ 0.038). Within males, MP was significantly lower than baseline starting at CS 1.25 for AI (P ≤ 0.040) and CS 2.25 for HiD (P ≤ 0.002). Within females, MP was significantly lower than baseline starting at CS 0.75 for AI (P ≤ 0.008) and CS 2.25 for HiD (P ≤ 0.001), with MP declining at a significantly later CS in HiD vs. AI females (CS x diet interaction P < 0.001). Data followed a sigmoidal relationship for males (AI males, r^2^ = 0.985; HiD males, r^2^ = 0.993; curves were not significantly different) and females (AI females, r^2^ = 0.981; HiD females, r^2^ = 0.983; curves were not significantly different). During disease progression, MP AUC negatively correlated with CS AUC for both AI (r = -0.380, P = 0.035) and HiD (r = -0.330, P = 0.087) mice (Figure 3B).

*Tibialis anterior weights*

Body weight-adjusted *tibialis anterior* weights positively correlated with age at CS2 for both AI (r = 0.662; P < 0.001) and HiD (r = 0.411; P = 0.090) mice (Figure 5A); for both AI males (r = 0.687, P = 0.014) and females (r = 0.625, P = 0.040), but not for HiD males and females. For every 1 mg/g body weight increase in *tibialis anterior* weight, AI and HiD mice experience a 10 d and 8 d delay in disease onset, respectively.

*Quadriceps weights*

Body weight-adjusted *quadriceps* weights positively correlated with age at CS2 for AI mice (r = 0.661, P < 0.001), but not for HiD mice (Figure 5B); for both AI males (r = 0.498, P = 0.100) and females (r = 0.887, P < 0.001). For every 1 mg/g body weight increase in *quadriceps* weight, AI and HiD mice experience a 3 d and 1 d delay in disease onset, respectively.

*Brain weights*

Body weight-adjusted brain weights were not significantly different between the diets (Figure 2C). Between the sexes, males had 17% lighter body weight-adjusted brain weights vs. females (P < 0.001). Body weight-adjusted brain weights positively correlated with age at CS4 (r = 0.986, P = 0.014; Figure 6A) and CS5 (r = 0.982, P = 0.018; Figure 6B). For every 1 mg/g body weight increase in brain weight, mice experience a 2 d delay in CS4 and a 2 d delay in CS5.
